# Detection of Pathogenic and Beneficial Microbes for Roselle Wilt Disease

**DOI:** 10.3389/fmicb.2021.756100

**Published:** 2021-11-01

**Authors:** Chih-Wei Wang, Yu-Hsiang Yu, Chun-Yao Wu, Ru-Ying Feng, Kshitij Tandon, Ying-Lien Chen, Sen-Lin Tang

**Affiliations:** ^1^Taitung District Agricultural Research and Extension Station, Council of Agriculture, Executive Yuan, Taitung, Taiwan; ^2^Biodiversity Research Center, Academia Sinica, Taipei, Taiwan; ^3^Molecular and Biological Agricultural Sciences, Taiwan International Graduate Program, Academia Sinica, Taipei, Taiwan; ^4^Graduate Institute of Biotechnology, National Chung Hsing University, Taichung, Taiwan; ^5^Department of Plant Pathology and Microbiology, National Taiwan University, Taipei, Taiwan; ^6^Master Program for Plant Medicine, National Taiwan University, Taipei, Taiwan; ^7^Biotechnology Center, National Chung Hsing University, Taichung, Taiwan

**Keywords:** rhizosphere, microbiome, roselle wilt disease, *Fusarium* wilt, *Fusarium solani*, *Bacillus velezensis*

## Abstract

Wilt disease of roselle (*Hibiscus sabdariffa* L.) is common in Taiwan; however, the causative agent remains unknown. The stems of wilted roselle are browned, slightly constricted, and covered by white aerial hyphae, suggesting that potential pathogens may originate from soil. To identify the potential pathogens, we conducted a rhizosphere microbiota survey in phenotypically healthy and diseased plants through fungal internal transcribed spacer (ITS) and bacterial 16S rRNA amplicon sequencing for uncovering the microbial compositions in the roselle rhizosphere. The fungal family Nectriaceae exhibited significantly higher abundance in diseased rhizospheres than in healthy rhizospheres, and this bacterial community was more specific to geography (i.e., plot-dependent) than to rhizosphere disease status. However, a few bacterial groups such as *Bacilli* were associated with the healthy rhizosphere. *Fusarium* species were the most dominant species of Nectriaceae in the survey and became the main target for potential pathogen isolation. We successfully isolated 119 strains from diseased plants in roselle fields. Koch’s postulates were used to evaluate the pathogenicity of these strains; our results indicated that *Fusarium solani* K1 (FsK1) can cause wilting and a rotted pith in roselles, which was consistent with observations in the fields. This is the first demonstration that *F. solani* can cause roselle wilt in Taiwan. Furthermore, these newly isolated strains are the most dominant operational taxonomic units detected in ITS amplicon sequencing in diseased rhizospheres, which serves as further evidence that *F. solani* is the main pathogen causing the roselle wilt disease. Administration of *Bacillus velezensis* SOI-3374, a strain isolated from a healthy roselle rhizosphere, caused considerable anti-FsK1 activity, and it can serve as a potential biocontrol agent against roselle wilt disease.

## Introduction

Roselle (*Hibiscus sabdariffa* L.) is a multipurpose crop that belongs to the *Malvaceae* family (Alshoosh, 1997; Islam et al., 2016). Roselle is native to West Africa and India and is currently grown in numerous tropical and subtropical regions. It is an annual or perennial, woody or herbaceous plant and can be divided into two common varieties that are cultivated for food (*H. sabdariffa* var. *sabdariffa*) and fiber (*H. sabdariffa* var. *altissima* Wester) (Alshoosh, 1997; Ansari et al., 2013). China and Thailand are the main growers of this crop in Asia; however, other regions including Mexico, Egypt, Senegal, Tanzania, Jamaica, and Mali are also major producers (Islam et al., 2016).

According to a national survey conducted by the Agricultural and Food Agency in Taiwan, as of 2019, the nationwide cultivation area of roselle was approximately 182.53 ha, with an annual yield of approximately 305,925 kg. Roselle grows at 18 to 35°C, optimally at 25°C, and is intolerant to low temperatures (Ansari et al., 2013). Several roselle cultivars are employed in Taiwan, among which Taitung No. 3 is the one with the highest yield, thickest calyx, and optimal processing quality (Chen and Chen, 2019). Various diseases occur in roselle plants in Taiwan, including gray mold (*Botrytis cinerea*), irregular leaf spot (*Cercospora malayensis*), anthracnose (*Colletotrichum gloeosporioides*), Sclerotinia rot (*Sclerotinia sclerotiorum*), Phytophthora infection (*Phytophthora nicotianae*), bacterial wilt (*Ralstonia solanacearum*), wrinkled leaves and phyllody disorder (16SrI phytoplasma), and diseases caused by nematodes and insect pests (Tzean, 2019).

Roselle wilt disease in Taiwan is prevalent from July to October, with a field incidence rate of up to 80%, leading to tremendous agricultural economic loss. Roselles are grown from seeds, and those that wilt do not flower, and they rot from the roots; the stems of wilted plants are browned, slightly constricted, and covered by white aerial hyphae. Rotted piths are also commonly found in the vertically dissected stem base. The causative agent of roselle wilt disease is yet to be identified in Taiwan, but it has been reported in several countries such as the United States, Malaysia, Mexico, Nigeria, and Egypt. For example, *F. oxysporum* causes vascular wilt on roselles in the United States and Malaysia (Ooi and Salleh, 1999; Ploetz et al., 2007); *Phytophthora parasitica* causes crown rot (black foot) along with necrosis at the bases of the stems as well as foliar wilt and death of roselles in Mexico (Estrada et al., 2001); *F. oxysporum* causes vascular wilt and stem blight on roselles in Nigeria (Amusa et al., 2005); *F. solani* and *Macrophomina phaseolina* cause roselle wilt; and *F. oxysporum* causes pre-emergence damping off on roselles in Egypt (Hassan et al., 2014).

The fungal genus *Fusarium* is a large pathogenic group in plants and animals. In this genus, *F. solani* is a common pathogen that causes root rot in many plants and causes aboveground symptoms such as wilt (Coleman, 2016). Sudden death syndrome in soybeans in North and South America (Westphal et al., 2008) and chili wilt disease in India (Sundaramoorthy et al., 2012) are well-known examples of plant diseases caused by *F. solani* infection. *F. solani* also causes complex diseases with other pathogens such as nematodes (Gomes et al., 2011).

Although numerous types of crops are infected and severely damaged by *Fusarium* species, effective disease control methods are limited. *Fusarium* species produce chlamydospores and thus survive in harsh environments (Hou et al., 2020); thus, infection with this species is challenging to control comprehensively. Along with the use of fungicides, the development of biological control (i.e., biocontrol) has been extensively studied in recent years. Biocontrol occurs naturally in soil environments, and its mechanisms can be categorized into three macro aspects: (1) competitive root colonization, (2) synthesis of allelochemicals, and (3) indirect plant growth promotion through induced systemic resistance (Borriss, 2020; Etesami and Adl, 2020). Biocontrol strains can be screened and isolated using diverse methods, with the microbiome survey having been employed in numerous microbiological and plant pathological studies (Benítez and Gardener, 2009; Poudel et al., 2016; O’Brien, 2017). Notably, the potential biocontrol strains and pathogens may inhabit the same space underground, and understanding the abundance and diversity of biocontrol microbes in healthy and diseased soils can provide mechanism-related insights regarding disease causes and opportunities for developing management strategies (Poudel et al., 2016). Numerous non-pathogenic bacteria and fungi have been used as biocontrol agents for disease management, including *Bacillus* spp., *Pseudomonas* spp., *Streptomyces* spp. and *Trichoderma* spp. (O’Brien, 2017). In Taiwan, 11 *Bacillus* strains (six *B. amyloliquefaciens* and three *B. subtilis* strains, one *B. mycoides*, and one *B. velezensis*) have been registered for plant disease control because *Bacillus* spp. form endospores that not only expand the shelf life of plant products but also exhibit strong control efficacy against various plant diseases (Borriss, 2020).

In this study, four main objectives were achieved: (1) fungal and bacterial communities were surveyed in the rhizospheres of healthy and diseased roselles; (2) we found that *Fusarium* species were overabundant in diseased roselle plants compared with their healthy counterparts; (3) the dominant fungal species of *F. solani* was successfully isolated and verified as being the most likely pathogen causing roselle wilt disease; (4) a bacterial strain, *Bacillus velezensis* SOI-3374, isolated from the healthy roselle rhizosphere exhibited a significant anti-*F. solani* effect *in vitro*, highlighting its potential as a biocontrol agent for this disease.

## Materials and Methods

### Sampling Sites and Soil Sample Collection

**FIGURE 1:**
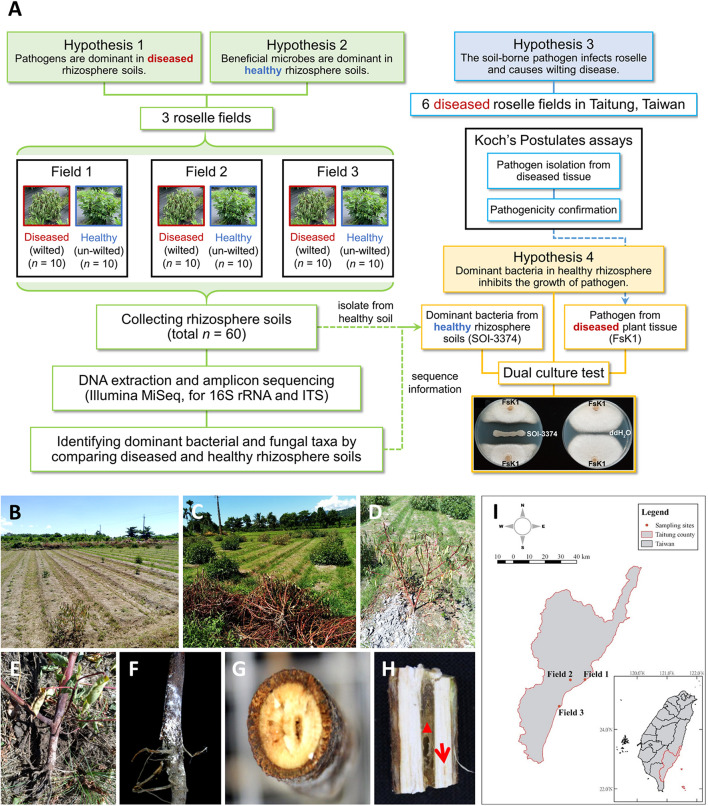
Schematic diagram of this study and the roselle wilt disease and symptoms found in Taitung, Taiwan. **(A)** A schematic diagram of the hypothesis and methodology. **(B)** and **(C)** Roselles were severely damaged by wilt disease. **(D)** Close-up shot of the wilting symptoms of roselle. **(E)** and **(F)** Stem base of wilted roselle colonized by fluffy, cotton-like aerial mycelia and tile mycelia with orange-white color. **(G)** Cross-section and **(H)** vertical dissection of diseased stem base, showing that the central pith (red triangle) was browning and rotten, with occasional lightly browning vascular bundles (red arrow). **(I)** Sampling sites of microbiome study in Taitung City (Field_01), Zhiben (Field_02), and Taimali (Field_03).

A schematic diagram of the hypothesis and methodology in this study was shown in Figure 1A. The experimental fields were located in Taitung City (Field_01; GPS: 22.745875°N; 121.150813°E), Zhiben (Field_02; GPS: 22.743759°N; 121.061361°E), and Taimali (Field_03; GPS: 22.581578°N; 120.993655°E) in southeastern Taiwan. There were no fertilizer used in these three fields. According to the information of Central Weather Bureau of Taiwan, the average temperature in July to October is 27.8°C (24.7°C for annual average); the average rainfall is 269.3 mm (144.8 mm for annual average), and the relative humidity is 75.3% (74.3% for annual average). From each field, rhizosphere soil samples of healthy (*n* = 10) and wilted (*n* = 10) roselle plants (*H. sabdariffa*) were collected, yielding 60 samples. Each selected plant was separated from others by over 2 m. The entire plant was dug out to collect the rhizosphere soil. After the loosely attached soil was removed, the soil tightly attached to roots was rapidly brushed out and collected in a 50-ml falcon tube. The rhizosphere soil samples were immediately placed on ice, transported to the laboratory, and stored at −80°C until DNA extraction.

### DNA Extraction, Marker Gene Amplification, Barcoding, and Sequencing

DNA extraction was performed using the DNeasy PowerSoil Kit (QIAGEN, MD, United States) according to the manufacturer’s protocol. For the bacterial composition survey, the V6 to V8 hypervariable region of 16S ribosomal RNA (rRNA) genes was amplified using polymerase chain reaction (PCR) with primers U968F and U1391R (Table 1), as previously described (Yang et al., 2016). All amplified and purified DNA were further added with specific barcodes according to the method of Yang et al. (2019). For the fungal composition survey, internal transcribed spacer (ITS) domain I of fungal rRNA genes was amplified using PCR with primers ITS1-F and ITS2 (Table 1), as previously described (Smith et al., 2018), with some modifications (Supplementary Material and Methods). Sixty bacterial and sixty fungal barcoded amplicon DNA samples were sent to Yourgene Bioscience (Taipei, Taiwan) and Tri-I Biotech (New Taipei City, Taiwan), respectively, for library construction and paired-end sequencing (2 × 300) on the Illumina MiSeq platform (Illumina, CA, United States). All of the bacterial and fungal community sequences were deposited in GeneBank (SRA accession PRJNA751843).

**TABLE 1:** Primers used in this study.

Primer	Use	Sequence (5′ to 3′)
JC889	16S rDNA forward (bacteria identification)	AGAGTTTGATCCTGGCTCAG
JC890	16S rDNA reverse (bacteria identification)	ACGGCTACCTTGTTACGACTT
JC1753	ITS4 (fungi identification)	TCCTCCGCTTATTGATATGC
JC976	ITS5 (fungi identification)	GGAAGTAAAAGTCGTAACAAGG
JC1189	TEF1α forward (fungi identification)	ATGGGTAAGGA(A/G)GACAAGAC
JC1190	TEF1α reverse (fungi identification)	GGA(G/A)GTACCAGT(G/C)ATCATGTT
JC2247	RPB2 forward (fungi identification)	GGGG(A/T)GA(C/T)CAGAAGAAGGC
JC2248	RPB2 reverse (fungi identification)	CCCAT(A/G)GCTTG(C/T)TT(A/G)CCCAT
JC2106	ITS1-F (microbiome study)	CTTGGTCATTTAGAGGAAGTAA
JC2107	ITS2 (microbiome study)	GCTGCGTTCTTCATCGATGC
U968F	16S rDNA forward (V6-V8 region, microbiome study)	AACGCGAAGAACCTTAC
U1391R	16S rDNA reverse (V6-V8 region, microbiome study)	ACGGGCGGTG(A/T)GT(A/G)C

### Bacterial 16S rRNA Gene Analysis

The raw reads were demultiplexed according to barcode into respective samples using Sabre^1^. On a per sample basis, R1 and R2 were first merged (–fastq_mergepairs), and then, the primers were removed (–search_pcr2) using USEARCH v11.0.667 (Edgar, 2010). The reads were further processed using MOTHUR v1.35.1 (Schloss et al., 2009) to retain high-quality reads that (1) had a length of 380–450 base pairs (bp), (2) contained homopolymers ≤ 8 bp, (3) did not have any ambiguous base, and (4) had an average Phred score of ≥ 20. Potential chimeras were identified (--uchime2_ref) and discarded (with options --mindiv 3 and --mode high_confidence) using USEARCH against the rdp_gold reference dataset^2^. To circumvent the memory limitation imposed by the 32-bit version of USEARCH, non-chimeric reads were clustered into operational taxonomic units (OTUs) by using VSEARCH v2.14.2 (Rognes et al., 2016) at a threshold of 97% identity, and OTU representative sequences were searched against the SILVA SSU Ref nr99 database v132 (Quast et al., 2013) using VSEARCH global alignment to identify the corresponding taxonomy of the best hit. Any OTU without a hit or with only a weak hit (i.e., an average percentage identity and percentage coverage < 93) was excluded. Finally, OTUs with the same affiliation were collapsed into the same OTU. Merging of OTUs that matched to the same reference sequence was conducted to avoid erroneous diversity inflation from sequences that were not well clustered.

### Fungal Internal Transcribed Spacer Domain Analysis

The raw reads were demultiplexed based on barcodes into the corresponding samples. On a per sample basis, the read merging and quality control steps were similar to those for bacterial amplicons, except for the high-quality reads defined as those that (1) had a length of 210–450 bp, (2) contained homopolymers ≤ 15 bp, (3) did not have any ambiguous base, and (4) had an average Phred score of ≥ 20. Potential chimeras were identified (–uchime2_ref) and discarded (with options –mindiv 3 and –mode high_confidence) using USEARCH against the UNITE reference dataset v7.2. Non-chimeric reads were clustered into OTUs by using VSEARCH at a threshold of 99.9% identity, and OTU representative sequences were searched against the UNITE database v7.2 (Nilsson et al., 2019) using VSEARCH global alignment to identify the corresponding taxonomy of the most suitable hit. Any OTU without a hit or with only a weak hit was excluded. Finally, OTUs with the same affiliation were also collapsed into the same OTU to avoid the erroneous diversity inflation from sequences not well clustered. The taxonomy of three OTUs were manually annotated using BLASTn search against the NCBI database (Supplementary Table 2).

### Wilted Roselle Sample Collection, Pathogen Isolation, and Growth Conditions

The roselle wilt disease samples were collected in July and October 2018 at six locations in Taitung, Taiwan: (1) Taitung District Agricultural Research and Extension Station (TDARES), (2) National Taitung University, (3) Beinan Township, (4) Donghe Township, (5) Dawu Township, and (6) Guanshan Township, and in October 2020 at TDARES. In total, 119 potential pathogens were isolated from infected tissues of roselles, including from the rotten root surface, constricted stem base, and browned pith. Tissues were cut and their surface sterilized for 1 min with 1% hypochlorous acid (repeated three times), washed with ddH_2_O, air dried, and placed on water agar (1% agar; BioShop, Burlington, ON, Canada). After incubation at 25°C for 3 d, the colonies of potential pathogens were purified and subcultured. Fungal strains were cultured on potato dextrose agar (PDA; 0.4% potato starch from infusion, 2% dextrose, and 1.5% agar; BioShop) at 28°C. Bacterial strains were cultured on nutrient agar (NA; 0.3% beef extract, 0.5% peptone, and 1.5% agar; BD Difco, Franklin Lakes, NJ, United States) at 30°C. All isolated fungal and bacterial strains (Supplementary Table 1) were stored at −80°C with 25% glycerol.

### Pathogen Morphology

The morphology of potential pathogens isolated from roselle samples was identified and observed using an inverted microscope (Olympus CKX53, Tokyo, Japan). A scanning electron microscope (SEM) was also used for sample observation (Supplementary Material and Methods).

### Phylogenetic Analysis

First, for identifying fungi, the 119 isolated strains (Supplementary Table 1) were preliminarily identified using the ITS with primers ITS4 and ITS5 (JC1753 and JC976, Table 1) (White et al., 1990); 16S rDNA with primers JC889 and JC890 (Table 1) was used to identify bacteria (Singh et al., 2013). To determine the phylogenetic relationships among the 107 isolated fungal strains (Supplementary Table 1), sequences were first compared using BLAST + software (Camacho et al., 2009), and those with > 99% identity (> 98% identity for oomycete strains) were identified as having the same sequence. Finally, 23 representative sequences were retrieved, and multiple sequence alignment was performed using the MAFFT online service (Talavera and Castresana, 2007). The poorly aligned positions and divergent regions in the alignment were eliminated using Gblocks (Talavera and Castresana, 2007). The maximum likelihood (ML) phylogeny was computed using IQ-TREE (v1.6.12) with the TNe + G4 model and 1000 bootstraps. A consensus tree was visualized and edited in iTOL (v4) (Letunic and Bork, 2019). To compare potential fungal pathogens isolated from diseased tissues with fungal OTU amplicons detected in rhizosphere soils, 23 representative sequences were searched against all the fungal OTU sequences by using BLAST. Among the 23 representative strains, the OTU sequences with identity > 97% and a length over 217 bp were retrieved. The abundance of each OTU was calculated from the average abundance of 60 samples from the OTU table after the OTUs were rarefied to the smallest sample size and OTUs less than 3 reads were removed; the abundance of each OTU was presented in < 10% of the samples. For further molecular identification, 13 sequences of potentially pathogenic *F solani* strains were analyzed with 32 sequences of *F. solani* strains from a recent study (Sandoval-Denis et al., 2019). Translation elongation factor 1-α (TEF1α, JC1189, and JC1190) (O’Donnell et al., 1998) and RNA polymerase II (RPB2, JC2247, and JC2248) (O’Donnell et al., 2008) sequences were used for further identifying these strains (Table 1). Phylogenetic trees were constructed on the basis of the ITS, TEF1α, and RPB2 sequences, and the phylogenetic relationship among fungal species was inferred using the ML method described previously (Talavera and Castresana, 2007; Camacho et al., 2009; Katoh et al., 2019; Letunic and Bork, 2019) with the TIM2e + I + G4 model.

### Koch’s Postulate and Disease Severity

*Fusarium solani* and *F. oxysporum* were prepared as a conidia suspension. Four 6-mm mycelium disks were punched from the PDA culture, added into 150 ml potato dextrose broth, and incubated at 28°C under 180 rpm for 8 to 10 d. The conidia were subsequently filtered using a two-layered Miracloth (EMD Millipore Corp., MA, United States) and collected by centrifugation at 3,500 rpm for 10 min, and the conidia were resuspended in sterile dH_2_O. The concentration of the freshly prepared conidia solution was calculated with a hemocytometer, and the solution was diluted for further tests. Koch’s postulate tests were conducted to identify potential pathogens of roselle wilt disease in Taiwan. The Taitung No. 3 roselle cultivar was used in this study. Three-week-old roselle roots were cut 5 cm from root tip with a sterile blade and then immersed into 30 ml ddH_2_O or 5 × 10^6^/ml of conidia suspension of *F. solani* K1 (GenBank accession number: MZ701961) for 30 min (Hou et al., 2020). After the inoculation, the roselles were planted in mixed soil (Silu Kudra peat moss: sandy loam [1:1]; Euler Humuswerk GmbH, Germany; Ming Sheng Industrial Co., Ltd., Pingtung, Taiwan), and 30 ml ddH_2_O or *Fusarium* was irrigated into the soil of each treatment, and roselle plants were grown in a growth chamber at 29°C. Five plants were used for each treatment, and a 16 h: 8 h, light: dark cycle was set for all treatments. During the period of pathogenicity tests, all plants were observed every day, and any symptom developed was recorded. The treatment temperature herein was set at 29°C, which is similar to the monthly mean temperature recorded in Taitung from July to August in 2018 according to the Taiwan Central Weather Bureau.

### Screening of Biocontrol Strains From Healthy Roselle Rhizosphere for Combating Roselle Wilt Fungus

Five grams of roselle rhizosphere soil (Field_03, No. H3) that was stored at −80°C was resuspended in 50 ml of sterilized water and heated for 30 min in a 65°C water bath. In total, 200 μL of the soil suspension was spread on NA medium and incubated in a 30°C incubator until a single colony appeared. Each single colony was streaked out, purified three times using the streaking method, and stored at −80°C. A confrontation assay was performed against the *F. solani* K1 (FsK1) strain on PDA medium. Strain SOI-3374, which showed the best antagonist effect was identified as *Bacillus velezensis* through 16S rRNA gene sequencing.

## Results

### Identification of Roselle Wilt Disease and Sampling

In this study, roselle plants were severely damaged by wilt disease in fields (Figures 1B,C). The symptoms included water loss in all leaves; fluffy, cotton-like aerial mycelia; and tile mycelia colonization on the stem base. The central pith exhibited browning and rotting (Figures 1D–H). To investigate the fungal and bacterial variations between diseased and healthy rhizospheres, roselles from three fields were selected for sampling, with each field being at least 8 km from the others (Figure 1I).

### Variations in Bacterial and Fungal Compositions in Roselle Rhizospheres

**FIGURE 2:**
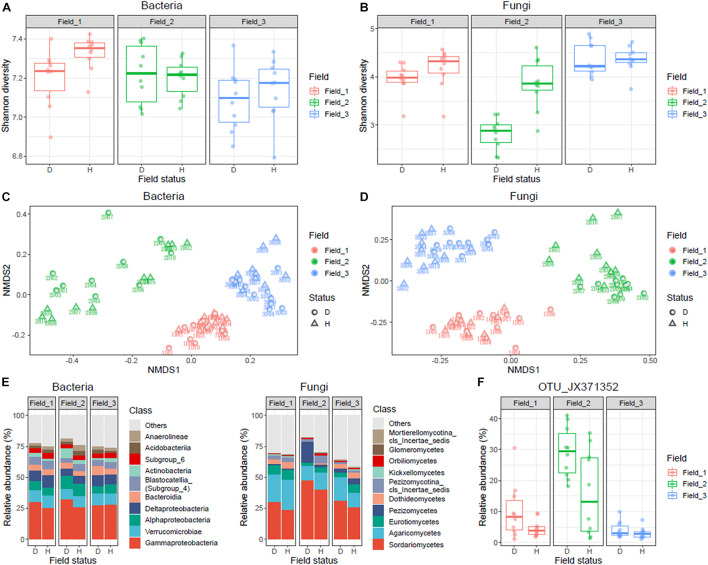
Statistical analysis and summary of bacterial and fungal communities in diseased and healthy roselle rhizosphere soils. Rhizosphere soil samples of healthy (*n* = 10) and wilted (*n* = 10) roselle plants were collected from three fields and a total of 60 samples were analyzed. **(A)** Shannon diversity of bacterial community. **(B)** Shannon diversity of fungal community. **(C)** nMDS analysis of bacterial OTUs. **(D)** nMDS analysis of fungal OTUs. **(E)** Bacterial and fungal community composition of the 10 most abundant classes on average. **(F)** The abundance distribution of fungal OTU_JX371352 across sampling fields. Field status, “D” for diseased and “H” for healthy.

In total, 53,352 bacterial and 6,725 fungal OTUs were identified from the 60 samples. Taxa not noted more than three times in at least 10% of the samples (*n* = 6) were excluded. After rarefaction, a total of 5,845 bacterial and 715 fungal OTUs were retained. The fungal Shannon diversity was significantly lower in diseased rhizosphere soils collected from Field_02 than in healthy samples (Figures 2A,B). Non-metric multidimensional scaling ordination indicated a marked difference between diseased and healthy rhizospheres in both fungal and bacterial communities (PERMANOVA, *p* < 0.05), although site-specific effects were noted (Figures 2C,D). Among the major fungal taxa, the fungal class Sordariomycetes had higher relative abundance in the diseased rhizosphere than in the healthy rhizosphere in all three fields (Figure 2E), and the fungal family Nectriaceae was dominant (Supplementary Figure 1); moreover, the OTU_JX371352 was the most abundant contributor to the differential abundance of Sordariomycetes between diseased and healthy rhizosphere soils (Figure 2F and Supplementary Table 2).

### Isolation of Potential Pathogens for Roselle Wilt Disease

**FIGURE 3:**
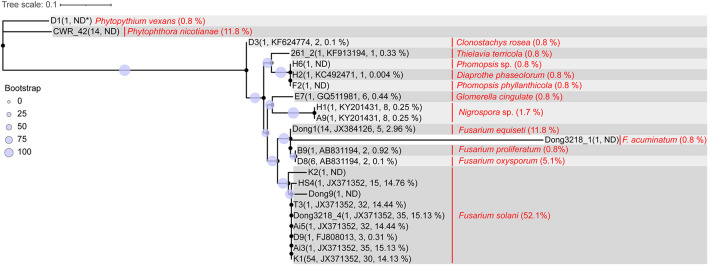
Sequence comparison of ITS1 region between isolated roselle wilt pathogens and rhizosphere amplicons. Parentheses following the strain ID indicate number of representative sequences, blasted most abundant OTU_ID, number of OTUs blasted, and sum of abundance (average percentage of the 60 samples). Words in red denote the species name of the isolate strain ID and parentheses indicate the percentage in 119 isolated strains. The maximum likelihood phylogeny was computed using IQ-TREE (v1.6.12) with the TNe + G4 model and 1000 bootstraps. A consensus tree was visualized and edited in iTOL (v4). (ND^∗^: the ITS sequences did not blast to any OTU amplicon sequence).

Three batches of wilted roselle samples were collected in July and October 2018 and in October 2020 in Taitung, Taiwan. A total of 119 isolates were obtained and initially verified using ITS (fungi) and 16S rDNA (bacteria) sequencing. These isolates included *F. solani* (52.1%, 62/119), *Phytophthora nicotianae* (11.8%, 14/119), *F. equiseti* (11.8%, 14/119), *F. oxysporum* (5.1%, 6/119), *F. acuminatum* (0.8%, 1/119), *F. proliferatum* (0.8%, 1/119), *Phytopythium vexans* (0.8%, 1/119), and other fungi or bacteria (16.8%, 20/119; Figure 3 and Supplementary Table 1).

### Sequence Comparison of Internal Transcribed Spacer 1 Regions Among Isolated Potential Pathogens and Rhizosphere Amplicons

To determine whether the isolated potential pathogens were correlated with rhizosphere amplicons, the 23 fungal ITS representative sequences (clustered from 107 isolates) were searched against the fungal OTU representative amplicons using BLAST. The results indicated that 56% (60/107) of the isolates were nearly perfect hits to OTU_JX371352 (with identity > 99% on the aligned length of approximately 221 bp), which belongs to the fungal species *F. solani*, a major cause of roselle wilt disease (Figure 3).

### Characterization of Prevalent Bacterial or Fungal Taxa in Healthy and Diseased Roselle Rhizospheres

The 10 most differentially abundant bacterial and fungal family taxa in diseased and healthy rhizosphere communities are listed in Table 2. In summary, the families Xanthomonadaceae, Microbacteriaceae, Enterobacteriaceae, Nectriaceae, and Ascobolaceae were more abundant in the diseased community, whereas Subgroup 6, Saccharimonadales, Anaerolineaceae, Bacillaceae, Chaetomiaceae, and Lasiosphaeriaceae were more abundant in healthy rhizosphere soils. In terms of genera, *Microbacteria*, *Luteolibacter*, *Pseudoxanthomonas*, and *Fusarium* were more abundant in diseased soils. Species belonging to *Bacillus* and *Cladorrhinum* were more abundant in healthy soils (Supplementary Table 3).

**TABLE 2:** Selected differential abundance of bacterial and fungal family taxa in healthy and diseased roselle rhizospheres.

	Diseased (D)				Healthy (H)			
		
		baseMean	log_2_Fold Change	*p*-value		baseMean	log_2_Fold Change	*p*-value
Bacterial community	f__Xanthomonadaceae	255.7	−2.0	3.98E-07	f__Subgroup_6	805.1	0.3	0.0027
	f__Microbacteriaceae	214.4	−1.7	2.13E-07	f__Saccharimonadales	322.2	0.4	0.0075
	f__Enterobacteriaceae	176.4	−2.2	4.70E-10	f__Anaerolineaceae	220.9	0.7	0.0049
	f__Rubritaleaceae	163.9	−1.2	6.46E-05	f__Bacillaceae	126.8	0.9	7.34E-05
	f__Flavobacteriaceae	133.3	−1.4	4.92E-05	f__Ktedonobacteraceae	124.7	1.8	0.0002
	f__Rhodanobacteraceae	108.7	−1.1	0.0012	f__SBR1031	123.9	0.6	0.0009
	f__Verrucomicrobiaceae	86.9	−0.7	0.0016	f__Thermoanaerobaculaceae	84.2	0.4	0.0087
	f__Caulobacteraceae	71.2	−0.6	0.0033	f__Methyloligellaceae	80.5	0.5	0.0028
	f__Bacteriovoracaceae	54.7	−0.8	0.0016	f__S0134_terrestrial_group	55.7	0.8	0.0035
	f__Promicromonosporaceae	51.4	−3.0	2.07E-09	f__Entotheonellaceae	53.0	0.6	0.0041
Fungal community	f__Nectriaceae	3956.1	−1.0	0.0012	f__Pezizomycotina_fam_ Incertae_sedis	545.6	0.9	0.0046
	f__Ascobolaceae	847.1	−3.3	5.12E-07	f__Chaetomiaceae	375.2	1.5	3.86E-06
	f__Hypocreales_fam_Incertae_sedis	334.6	−1.4	0.0005	f__Lasiosphaeriaceae	348.6	1.7	0.0009
	f__unidentified_o__Hypocreales	290.7	−1.8	9.66E-07	f__unidentified_o__Branch06	208.1	1.5	0.0054
	f__unidentified_c__Sordariomycetes	132.5	−2.2	1.47E-05	f__Pyronemataceae	157.0	1.7	0.019
	f__unidentified_o__Xylariales	19.4	−1.8	0.0013	f__Sordariaceae	80.2	2.8	2.22E-05
	f__Phaeosphaeriaceae	18.4	−1.6	3.32E-05	f__Ustilaginaceae	51.0	1.3	0.0015
	f__Marasmiaceae	9.4	−1.7	0.0019	f__Lophiostomataceae	49.2	1.6	0.0058
	f__Amphisphaeriaceae	5.9	−2.1	7.03E-06	f__Acaulosporaceae	34.4	2.5	0.0001
	f__Dothideomycetes_fam_Incertae_ sedis	5.8	−1.2	0.0024	f__unidentified_o__Sordariales	28.0	2.6	3.64E-09

### *Fusarium solani* Causes Roselle Wilt Disease

**FIGURE 4:**
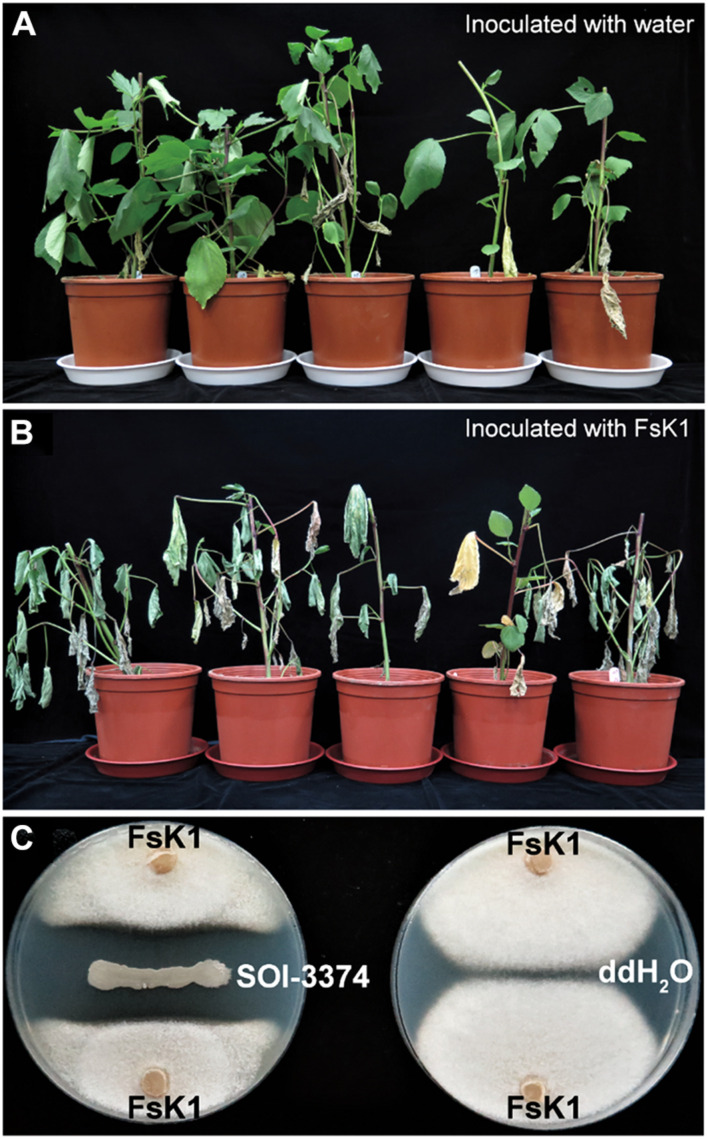
*Fusarium solani* K1 (FsK1) can cause roselle wilt disease. **(A)** Three-week-old roselles remained healthy after inoculation with ddH_2_O. **(B)** Those inoculated with the conidia of FsK1 exhibited wilting symptoms. **(C)** SOI-3374 showed antagonistic activity against FsK1 compared to ddH_2_O control.

**FIGURE 5:**
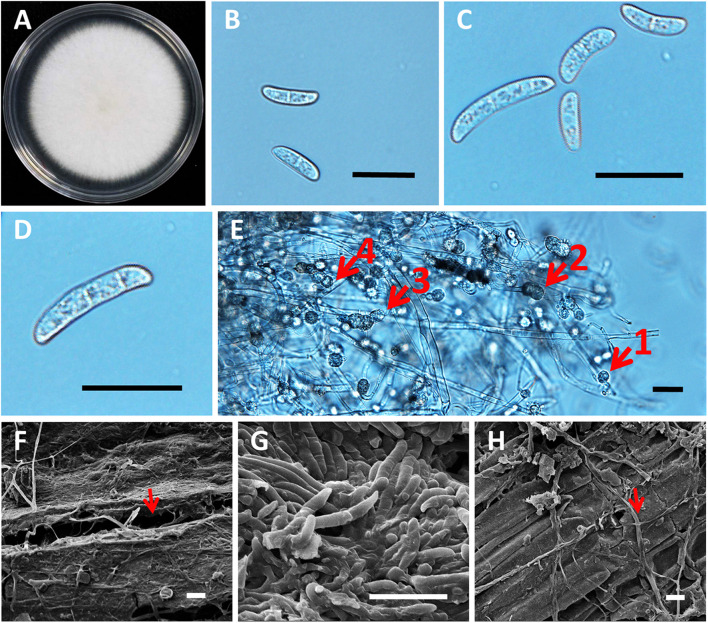
Morphology of roselle wilt fungus, FsK1. **(A)** Colony on PDA medium with white to light cream color, irregular margins, and sparse aerial mycelia. **(B)** Microconidia were 13.46 ± 3.52 μm × 4.46 ± 0.81 μm, hyaline, oval to ellipsoid, with zero to one septum. **(C)** Microconidia and macroconidia. **(D)** Macroconidia were 30.9 ± 7.12 μm × 5.95 ± 1.52 μm, hyaline, straight to falciform with three to four septa, whereas apical cells were blunt, and the foot shape of the basal cell was poorly developed. **(E)** Chlamydospores were 10.38 μm ± 2.94 μm, hyaline or dark brown, oval to globose, one to four cells growing intercalary or terminally on the hyphae, and usually with oil droplets. The numbered red arrows indicate 4 different clusters with 1 to 4 chlamydospores grown on a single chain of the hyphae. **(F)** Opening in the root surface with hyphae (red arrow). **(G)** Surface of the stem base was colonized with hyphae and conidia of *Fusarium* spp. **(H)** Hyphae (red arrow), but not conidia, were observed in the rotten pith. Scale bar: 20 μm.

Koch’s postulates were applied to verify the pathogenicity of our isolates to roselle wilt disease. Five to seven days after inoculation (dpi), conidia of FsK1 caused roselle wilt on 3-week-old roselles. The same symptoms were observed in both inoculation tests and fields (Figures 4, 5). The microbes reisolated from the FsK1-infected roselles were further identified as *F. solani* species complex by using ITS, TEF1α, and RPB2 sequence comparison. To the best of our knowledge, this is the first demonstration that *F. solani* species complex is the causative agent of roselle wilt disease in Taiwan. By contrast, isolated microbes other than *F. solani* (e.g., *F. oxysporum*) only caused leaf yellowing (Supplementary Figure 2). In virulence testing, another *F. solani* strain, K2, (isolated from the rotten pith of wilted roselle) in the field also caused roselle wilt in the inoculation test with conidia (Supplementary Figure 3).

### Phylogenetic Analysis and Morphology Observation of *Fusarium solani* K1

**FIGURE 6:**
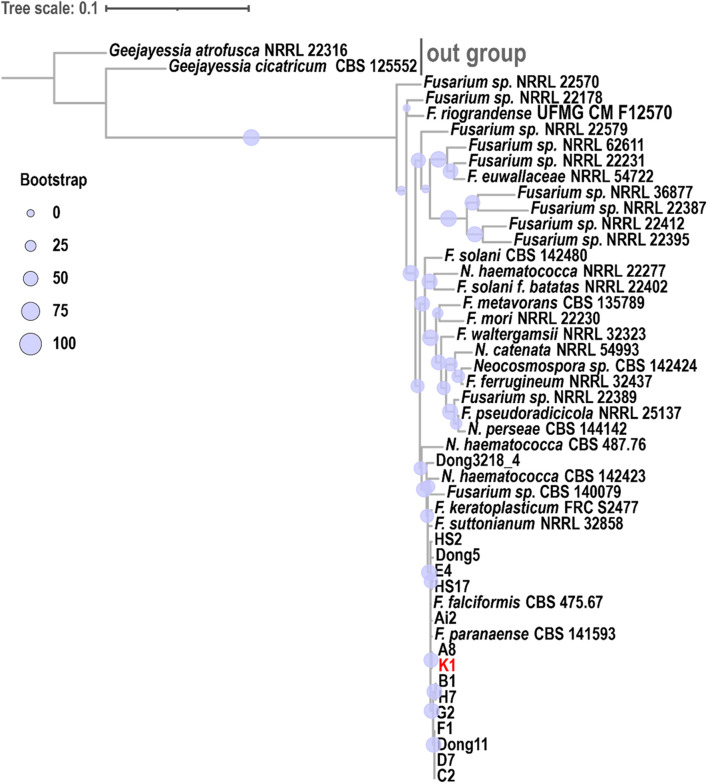
Phylogenetic analysis of *Fusarium solani* K1 (FsK1). A phylogenetic tree was constructed using ITS, TEF1α, and RPB2 sequences. FsK1 (K1 in the figure) exhibited the closest relationship with *F. paranaense* and *F. falciformis*, both belonging to the *F. solani* species complex. The phylogenetic relationship among fungal species was inferred using the maximum likelihood method with the TIM2e + I + G4 model and 1000 bootstraps. A consensus tree was visualized and edited in iTOL (v4).

The phylogenetic trees of FsK1 were constructed using ITS, TEF1α, and RPB2 sequences (Figure 6). Most of the 13 isolated strains were grouped together, including FsK1, which had the closest relationship with *F. paranaense* CBS 141593 and *F. falciformis* CBS 475.67, both of which belong to the *F. solani* species complex (Hypocreales, Nectriaceae) (Sandoval-Denis et al., 2019). The reference strain *F. paranaense* CBS 141593 was isolated from soybeans in Brazil, and the strain *F. falciformis* CBS 475.67 originated from human mycetoma in Puerto Rico (Sandoval-Denis et al., 2019). The morphology of FsK1 was observed under an optical microscope. The FsK1 colony on the PDA medium was white to cream color with irregular margins and sparse aerial mycelia (Figure 5A). The microconidia of FsK1 were 13.46 ± 3.52 μm × 4.46 ± 0.81 μm, hyaline, and oval to ellipsoid, with zero to one septum, and conidiogenous cells were monophalides with abundant growth and false heads (Figures 5B,C). The macroconidia of FsK1 were 30.9 ± 7.12 μm × 5.95 ± 1.52 μm, hyaline, and straight to falciform with 3-4 septa, whereas apical cells were blunt, and the foot shape of the basal cell was poorly developed (Figure 5C,D). The chlamydospores of FsK1 were 10.38 ± 2.94 μm and hyaline or dark brown; they contained one to four cells, were round to globose, and exhibited growth intercalary in or terminally on the hyphae, with a thick and smooth cell wall (Figure 5E). SEM observation revealed openings on the root surface with hyphae (red arrow, Figure 5F), and the surface of the stem base was colonized with hyphae and conidia of *Fusarium* spp. (Figure 5G). By contrast, hyphae (red arrow), but not conidia, were observed in the rotten pith (Figure 5H).

### *Bacillus velezensis* SOI-3374 Has the Potential to Control Roselle Wilt Disease

Confrontation assay of bacteria isolated from healthy rhizosphere soil (Field_03) was conducted. Among hundreds of strains we isolated, strain SOI-3374 has the best antagonistic effects toward FsK1. The clear inhibition zone which FsK1 could not grow was formed between the two microorganisms on the plate (Figure 4C).

## Discussion

In this study, amplicon sequencing was used to explore the mycobiota and microbiota in healthy and diseased roselle rhizosphere soils; the results indicated that the abundant fungal OTU, OTU_JX371352 was considerably more common in diseased soil rhizospheres than in healthy soil rhizospheres and is thus a potential pathogen. Through pathogen isolation experiments, *F. solani* was isolated from the diseased roselle tissues with a high proportion of 52.1% (Figure 3 and Supplementary Table 1). Among the strains, FsK1 and *F. solani* K2 were pathogenic to roselles, as confirmed by Koch’s postulates. Moreover, according to the high *Bacillus* population in healthy soil samples, the *B. velezensis* SOI-3374 isolated from the healthy soil samples was verified to prevent FsK1 growth *in vitro*.

### Community Variation Between Diseased and Healthy Roselle Rhizospheres

In our study, compared with healthy fields, all diseased fields had a higher abundance of pathogenic *F. solani*, suggesting that *F. solani* is the major causative agent of roselle wilt disease. In addition to *F. solani* (the Nectriaceae family), abundant populations of Xanthomonadaceae, Microbacteriaceae, Enterobacteriaceae, Flavobacteriaceae, Rubritaleaceae, and Ascobolaceae were detected in diseased soils. By contrast, large populations of Saccharimonadales, Subgroup 6, Bacillaceae, Anaerolineaceae, Chaetomiaceae, and Lasiosphaeriaceae were observed in healthy soils. In a related study investigating healthy or *Fusarium*-wilt-diseased soils, Yuan et al. found that *F. oxysporum*, Xanthomonadaceae, Bacillaceae, and *Gibberella* were more abundant in diseased soils, whereas more populations of Bradyrhizobiaceae, Comamonadaceae, *Mortierella*, *Streptomyces mirabilis*, and non-pathogenic *Fusarium* were observed in healthy soils (Yuan et al., 2020). In another study, Liu and Zhang compared healthy and *Fusarium*-diseased soils of cucumber fields and found that in the soil fungal community, Blastocladiomycota and *Mycothermus* were significantly more abundant in healthy soils and played an influential role in disease development (Liu and Zhang, 2021). In line with the findings of these studies, our results demonstrated that Blastocladiaceae (belonging to Blastocladiomycota) and Chaetomiaceae (containing the *Mycothermus* genus) were considerably more abundant in healthy roselle rhizosphere soils, whereas Xanthomonadaceae and pathogenic *Fusarium* were strongly enriched in diseased soils. *Mycothermus* has been reported as a potential pathogen-suppressive microorganism with an inverse proportion to the abundance of *Fusarium* (Huang et al., 2019), which may serve as a proxy to determine the risk probability of *Fusarium* diseases. Furthermore, the discovery of abundant Firmicutes in healthy soils (Liu and Zhang, 2021) agrees with our observation of abundant Bacillaceae (belonging to Firmicutes) in healthy soils. Within the family Bacillaceae, the *Bacillus* genus is a well-known bacterial group that has been applied in fields as biological control agents, and it is a taxon with a contrasting abundance between healthy and diseased rhizosphere soils (Beneduzi et al., 2012; Borriss, 2020).

Although a plethora of evidence suggests a connection between microbial (including fungal and bacterial) communities and roselle wilt disease, technical limitations emerge when ITS1F and ITS2 primers are used to differentiate pathogenic or non-pathogenic *Fusarium* species unless accompanied with isolation and inoculation tests. The primer set is widely used in studies of *Fusarium* diseases, which have explored the resolution of universal primers; however, more evaluation is required in the future (Tedersoo et al., 2016; Liu and Zhang, 2021).

### The Most Abundant *Fusarium solani* Strains Cause Roselle Wilt Disease

In this study, *F. solani*, and not *F. oxysporum*, was the main causative agent of roselle wilt disease (Figure 4). Different *F. solani* strains, K1 and K2, caused roselle wilt similar to that found in fields (Figure 4 and Supplementary Figure 3). In general, *F. oxysporum* exists in the vascular tissues and causes wilt, whereas *F. solani* exists in the crown or root and causes root rot (Westerlund et al., 1974; Trapero Casas and Jiménez Díaz, 1985; Ales and Lenka, 1997). Only five *F. oxysporum* strains were noted among our 119 isolates; thus, these five *F. oxysporum* strains are highly likely to be involved in secondary infection. Moreover, *F. solani* likely infects or colonizes the pith, vascular tissues, or both, which is supported by the following studies: (1) a histochemical study of yellow poplar revealed that *F. solani* infected the pith tissue and caused pith rot symptoms in plants (Arnett and Witcher, 1974); (2) in China, physic nut infected by *F. solani* caused root rot and pith rot symptoms (Wu et al., 2011); (3) yellowing and wilting symptoms of cannabis plants were observed in northern California, with *F. solani* causing pith tissue browning and plant death within 6 to 10 wk after inoculation (Punja et al., 2018); and (4) in passion fruits, *F. solani* caused the vascular bundle browning of plants (Cole et al., 1992). In a phylogenetic study, the reference strain *F. paranaense* CBS 141593 (*F. solani* species complex, close relationship to the K1 strain) was isolated from soybean, which is consistent with the finding of a previous study that *F. solani* causes symptoms such as sudden death syndrome in soybean (Westphal et al., 2008); we also suggest that the pathogenic strains isolated in the current study are closely related to the phytopathogenic *F. solani* species complex. Although the strain *F. falciformis* CBS 475.67 is closely related to the strains we isolated in this study, it originates from human mycetoma. The relationship between human pathogens and plant pathogens should be further studied to confirm whether human pathogens infect humans only or plants as well.

### *Bacillus* Has Potential Control Effects in Roselle Wilt Disease

Regarding the development of control strategies for roselle wilt disease, *Bacillus velezensis* SOI-3374, isolated from the healthy roselle rhizosphere, can control roselle wilt, as demonstrated in confrontation assay. In general, chitin-degrading and other cell wall–degrading enzymes may act as crucial substances that enable *Bacillus* to inhibit *Fusarium*, whose cell wall is composed of α- and β-1, 3-glucans (Schoffelmeer et al., 1999; Khan et al., 2018). The biocontrol activity of *Bacillus* species against plant-pathogenic *F. solani* has been documented. For example, a *Bacillus* commercial formulation effectively controlled the crown and root rot of tomato caused by *F. solani* in field trials (Pastrana et al., 2016); *B. subtilis* reduced the damping off incidence of cucumber seedlings caused by *F. solani* (Al-Fadhal et al., 2019).

A recent study indicated that *B. velezensis* effectively prevented *F. solani* infection in passion fruits (Wang et al., 2021). *B. velezensis* strains with secondary-metabolite-secretion ability could inhibit *F. solani* and oomycetes, and these strains were reported to serve plant growth–promoting rhizobacteria (Cheffi et al., 2019). Furthermore, in a rhizosphere study, *B. velezensis* that inhabited the grass (*Sporobolus airoides*) rhizosphere was isolated and the whole genome sequenced; this *B. velezensis* strain exhibited strong antifungal activity against phytopathogens that cause root rot, including *F. solani*, *F. oxysporum*, *Phytophthora capsici*, and *Rhizoctonia solani* (Martínez-Raudales et al., 2017). Although *B. velezensis* holds promise for controlling plant pathogens, the mechanisms through which *B. velezensis* SOI-3374 controls roselle wilt remain to be explored.

## Data Availability Statement

Data were available within the article or its Supplementary Material. All of the bacterial and fungal community sequences were deposited in GeneBank (SRA accession PRJNA751843).

## Author Contributions

C-WW, Y-HY, Y-LC, and S-LT planned and designed the research. C-WW, Y-HY, C-YW, and R-YF performed experiments, conducted fieldwork, analyzed data etc. KT analyzed the sequence data. C-WW, Y-HY, Y-LC and S-LT wrote the manuscript. All authors contributed to the article and approved the submitted version.

## Conflict of Interest

The authors declare that the research was conducted in the absence of any commercial or financial relationships that could be construed as a potential conflict of interest.

## Publisher’s Note

All claims expressed in this article are solely those of the authors and do not necessarily represent those of their affiliated organizations, or those of the publisher, the editors and the reviewers. Any product that may be evaluated in this article, or claim that may be made by its manufacturer, is not guaranteed or endorsed by the publisher.

## Footnotes

1
https://github.com/najoshi/sabre

2
http://drive5.com/uchime/rdp_gold.fa
