# Supplementary material for: Detection of Pathogenic and Beneficial Microbes for Roselle Wilt Disease
Source: Front Microbiol. 2021 Nov 1;12:756100. doi: 10.3389/fmicb.2021.756100 (PMC8591290; doi:10.3389/fmicb.2021.756100)
Supplement: Supplementary file 1 [file Data_Sheet_1.zip › Supplementary figures.docx]

**Supplementary figures**

**Figure S1**

**
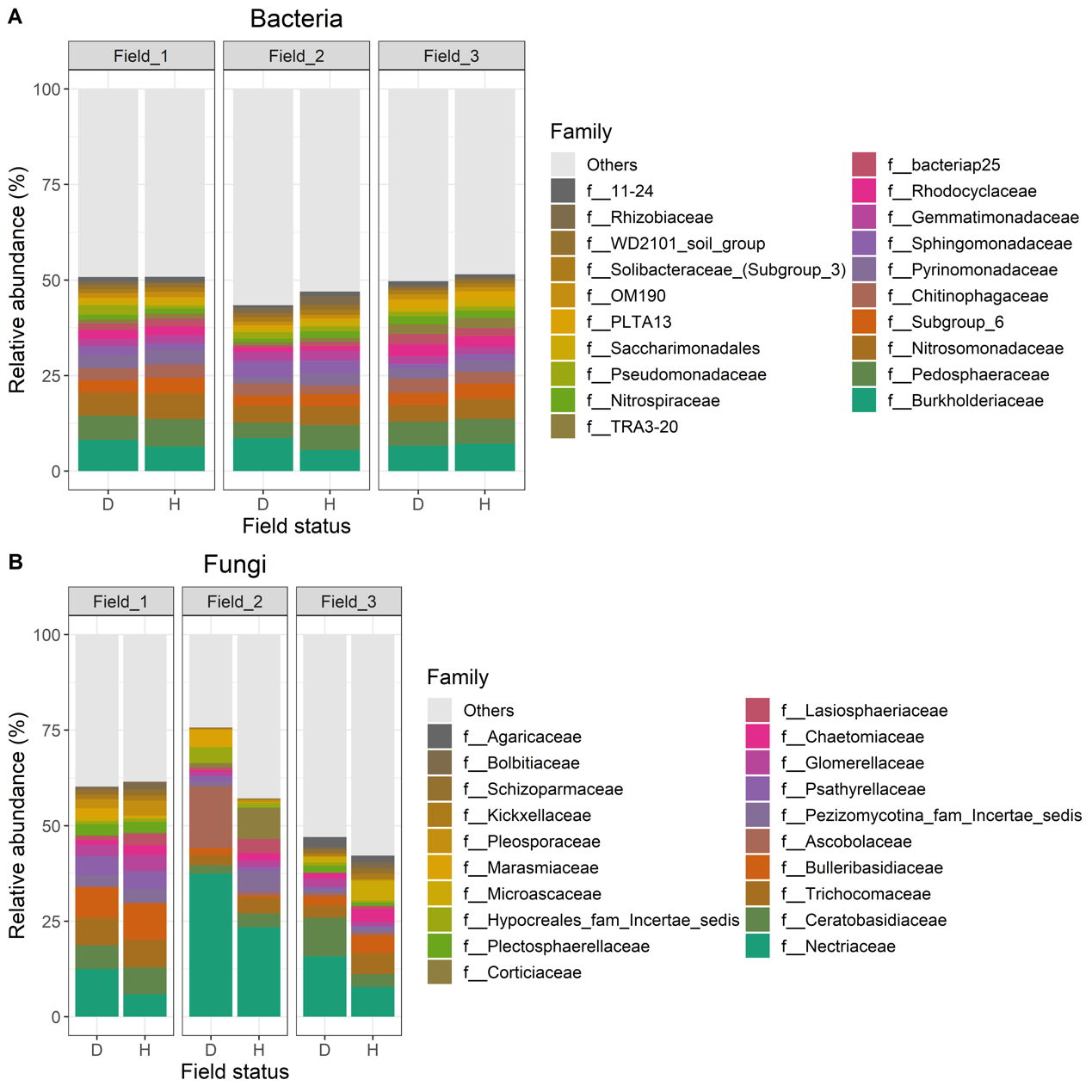
**

**Figure S1** Bacterial and fungal community composition of the 20 most abundant families on average.

**Figure S2**


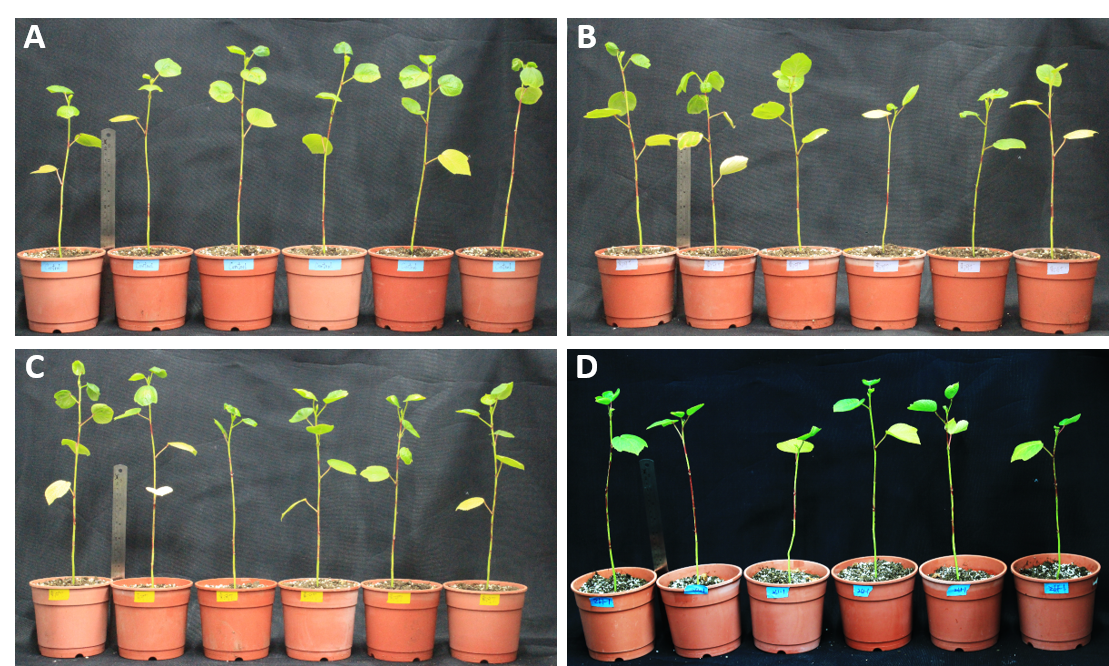


**Figure S2 *F. oxysporum* caused leaf-yellowing symptom on roselles.** **(A)** Inoculation with ddH_2_O as a control, no symptoms occurred. After inoculation with 5 × 10^6^ conidia/ml suspension of **(B)** strain DH5, **(C)** strain DH7, and **(D)** strain 261-1, only a few yellowing leaves were observed but no wilt symptoms.

**Figure S3**


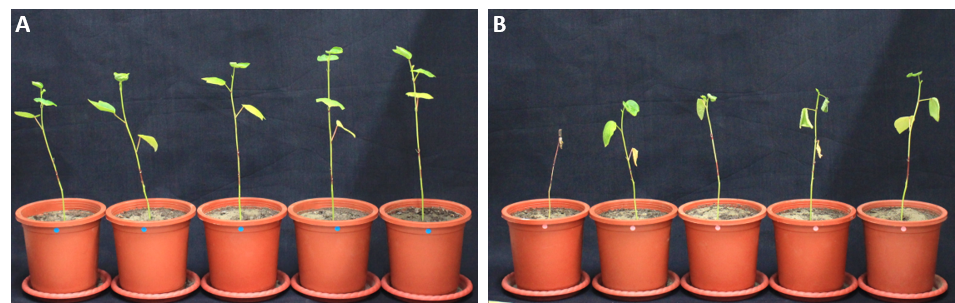


**Figure S3 *F. solani* K2 (FsK2) can also cause roselle wilt. (A)** Inoculation of ddH_2_O served as control. Virulence testing of FsK2 **(B)**, which was also isolated from a rotten pith of the diseased roselle sample, indicated that four of five plants were beginning to wilt after inoculation with 30 ml of 5 × 10^6^ conidia/ml of conidia suspension.
