# Supplementary material for: Detection of Pathogenic and Beneficial Microbes for Roselle Wilt Disease
Source: Front Microbiol. 2021 Nov 1;12:756100. doi: 10.3389/fmicb.2021.756100 (PMC8591290; doi:10.3389/fmicb.2021.756100)
Supplement: Supplementary file 1 [file Data_Sheet_1.zip › Supplementary information.docx]

**Supplementary information**

**Supplementary materials and methods**

**Amplification of the ITS domain of fungal rDNA and sequencing**

For amplification of the ITS domain of fungal rDNA, we used ITS1-F primers (5′-CTT GGT CAT TTA GAG GAA GTA A -3′) and ITS2 (5′-GCT GCG TTC ATC GAT GC -3′). For each sample, a 25-μl polymerase chain reaction (PCR) mix was prepared containing 2 ng DNA template, 5X KAPA HiFi Buffer, 10 mM KAPA dNTP Mix, 1 U/μL KAPA HiFi DNA Polymerase (KAPA Biosystem, Boston, USA), and 0.3 μM of each primer (Tri-I, New Taipei, Taiwan). The PCR reaction conditions consisted of an initial 95 ℃ for 3 min followed by 25 cycles of 98 ℃ for 20 sec, 57.5 ℃ for 20 sec, and 72 ℃ for 20 sec, and a final extension of 72 ℃ for 3 min. Subsequently, DNA tagging PCR (five cycles) was used to tag each of the PCR products (every six samples were tagged individually and mixed, resulting in 10 mixtures for 60 samples) of the fungal internal transcribed spacer (ITS) region. The PCR products were run in 2% agarose gel (SeaKem LE Agarose, Lonza, ME, USA), purified with a MinElute Gel Extraction kit (Qiagen, Hilden, Germany), and quantified using a QuantiFluor dsDNA System (Promega Corporation, Madison, WI, USA) on a Qubit 2.0 Fluorometer (Invitrogen, Grand Island, NY). The paired-end library was constructed with a Celero DNA-Seq System (1-96) (Nugen, San Carlos, CA, USA); all procedures were in accordance with respective manufacturers’ instructions. The library concentration and quality were assessed on a Bioanalyzer 2100 (Agilent Technologies, Santa Clara, CA, USA) using a DNA 1000 lab chip (Agilent Technologies). ITS amplicon libraries were sequenced 2 × 301 + 16 bp (dual index) by using a Miseq Reagent kit v3 (600 cycles) on an Illumina MiSeq system, following the manufacturer’s instructions.

**Scanning electron microscopy**

Wilted roselle plants from fields and Koch’s postulate tests were observed under a scanning electron microscope (SEM). The stem bases of wilted roselles were cut into 5 × 5 mm^2^ sections and simply rinsed with 75% ethanol for 20 min (repeated three times); subsequently, the samples were dried overnight in a 60 °C electronic incubator (Kwang Shen KS-31, Taiwan). The dried samples were mounted on metal stubs for gold coating with an ion coater (Eiko Engineering, Japan). Samples were then observed and photographed under a SEM (FEI Inspect S, USA), using an accelerating voltage of 1.5 kV. These instruments were provided by the Technology Commons, College of Life Science, National Taiwan University.
