# Supplementary material for: Detection of Pathogenic and Beneficial Microbes for Roselle Wilt Disease
Source: Front Microbiol. 2021 Nov 1;12:756100. doi: 10.3389/fmicb.2021.756100 (PMC8591290; doi:10.3389/fmicb.2021.756100)
Supplement: Supplementary file 1 [file Data_Sheet_1.zip › Supplementary tables.docx]

**Supplementary tables**

| **Table S1. Strains isolated from diseased roselle samples in Taitung, Taiwan** | | | | | |
| --- | --- | --- | --- | --- | --- |
| Isolate No. | Strain | Species^a^ | Isolation part | Collection location | Collection date |
| 1 | Dong1 | *Fusarium equiseti* | ---^b^ | NTTU^c^ | 2018/07/06 |
| 2 | Dong3 | *Fusarium solani* | --- | NTTU | 2018/07/06 |
| 3 | Dong4 | *Fusarium equiseti* | --- | NTTU | 2018/07/06 |
| 4 | Dong5 | *Fusarium solani* | --- | NTTU | 2018/07/06 |
| 5 | Dong7 | *Fusarium equiseti* | --- | NTTU | 2018/07/06 |
| 6 | Dong8 | *Fusarium equiseti* | --- | NTTU | 2018/07/06 |
| 7 | Dong9 | *Fusarium solani* | --- | NTTU | 2018/07/06 |
| 8 | Dong10 | *Fusarium solani* | --- | NTTU | 2018/07/06 |
| 9 | Dong11 | *Fusarium solani* | --- | NTTU | 2018/07/06 |
| 10 | Dong12 | *Fusarium solani* | --- | NTTU | 2018/07/06 |
| 11 | Dong13 | *Fusarium solani* | --- | NTTU | 2018/07/06 |
| 12 | Dong14 | *Fusarium solani* | --- | NTTU | 2018/07/06 |
| 13 | Dong15 | *Fusarium equiseti* | --- | NTTU | 2018/07/06 |
| 14 | 261-1 | *Fusarium oxysporum* | --- | Beinan | 2018/07/27 |
| 15 | 261-2 | *Thielavia terricola* | --- | Beinan | 2018/07/27 |
| 16 | Dong3218-1 | *Fusarium acuminatum* | --- | NTTU | 2018/07/27 |
| 17 | Dong3218-4 | *Fusarium solani* | --- | NTTU | 2018/07/27 |
| 18 | T2 | *Fusarium solani* | --- | NTTU | 2018/07 |
| 19 | T3 | *Fusarium solani* | --- | NTTU | 2018/07 |
| 20 | DH1 | *Phytophthora nicotianae* | --- | Donghe | 2018/07/18 |
| 21 | DH2 | *Phytophthora nicotianae* | --- | Donghe | 2018/07/18 |
| 22 | DH5 | *Fusarium oxysporum* | --- | Donghe | 2018/07/18 |
| 23 | DH6 | *Fusarium oxysporum* | --- | Donghe | 2018/07/18 |
| 24 | DH7 | *Fusarium oxysporum* | --- | Donghe | 2018/07/18 |
| 25 | DH8 | *Fusarium solani* | --- | Donghe | 2018/07/18 |
| 26 | DH9 | *Fusarium solani* | --- | Donghe | 2018/07/18 |
| 27 | DH10 | *Fusarium solani* | --- | Donghe | 2018/07/18 |
| 28 | Ai1 | *Fusarium solani* | --- | Aiguopu | 2018/07/06 |
| 29 | Ai2 | *Fusarium solani* | --- | Aiguopu | 2018/07/06 |
| 30 | Ai3 | *Fusarium solani* | --- | Aiguopu | 2018/07/06 |
| 31 | Ai4 | *Fusarium solani* | --- | Aiguopu | 2018/07/06 |
| 32 | Ai5 | *Fusarium solani* | --- | Aiguopu | 2018/07/06 |
| 33 | HS1 | *Phytophthora nicotianae* | --- | Hongshi | 2018/07/18 |
| 34 | HS2 | *Fusarium solani* | --- | Hongshi | 2018/07/18 |
| 35 | HS4 | *Fusarium solani* | --- | Hongshi | 2018/07/18 |
| **Table S1. continued** | | | | | |
| 36 | HS5 | *Phytophthora nicotianae* | --- | Hongshi | 2018/07/18 |
| 37 | HS6 | *Phytophthora nicotianae* | --- | Hongshi | 2018/07/18 |
| 38 | HS7 | *Phytophthora nicotianae* | --- | Hongshi | 2018/07/18 |
| 39 | HS8 | *Phytophthora nicotianae* | --- | Hongshi | 2018/07/18 |
| 40 | HS9 | *Phytophthora nicotianae* | --- | Hongshi | 2018/07/18 |
| 41 | HS10 | *Phytophthora nicotianae* | --- | Hongshi | 2018/07/18 |
| 42 | HS11 | *Phytophthora nicotianae* | --- | Hongshi | 2018/07/18 |
| 43 | HS12 | *Phytophthora nicotianae* | --- | Hongshi | 2018/07/18 |
| 44 | HS13 | *Phytophthora nicotianae* | --- | Hongshi | 2018/07/18 |
| 45 | HS14 | *Phytophthora nicotianae* | --- | Hongshi | 2018/07/18 |
| 46 | HS15 | *Fusarium solani* | --- | Hongshi | 2018/07/18 |
| 47 | HS16 | *Fusarium solani* | --- | Hongshi | 2018/07/18 |
| 48 | HS17 | *Fusarium solani* | --- | Hongshi | 2018/07/18 |
| 49 | 2010-1 | *Bordetella avium* | --- | Beinan | 2018/07/27 |
| 50 | 2010-2 | *Bacillus thuringiensis* | --- | Beinan | 2018/07/27 |
| 51 | Dong3218-3 | *Achromobacter xylosoxidans* | --- | NTTU | 2018/07/27 |
| 52 | Dong2 | *Enterobacter cloacae* | --- | NTTU | 2018/07/06 |
| 53 | Dong6 | *Enterobacter cloacae* | --- | NTTU | 2018/07/06 |
| 54 | T8 | *Bosea* sp. | --- | NTTU | 2018/07 |
| 55 | T11 | *Brevundimonas* sp. | --- | NTTU | 2018/07 |
| 56 | T13 | *Sphingobacterium* sp. | --- | NTTU | 2018/07 |
| 57 | T15 | *Stenotrophomonas* sp. | --- | NTTU | 2018/07 |
| 58 | T16 | *Achromobacter* sp. | --- | NTTU | 2018/07 |
| 59 | T17 | *Sphingobacterium thalpophilum* | --- | NTTU | 2018/07 |
| 60 | T18 | *Achromobacter* sp. | --- | NTTU | 2018/07 |
| 61 | K1 | *Fusarium solani* | --- | TDARES^d^ | 2018/10/25 |
| 62 | K2 | *Fusarium solani* | --- | TDARES | 2018/10/25 |
| 63 | K3 | *Fusarium equiseti* | --- | TDARES | 2018/10/25 |
| 64 | CWR-42 | *Phytophthora nicotianae* | --- | TDARES | 2018/02/05 |
| 65 | A8 | *Fusarium solani* | pith | TDARES | 2020/10/19 |
| 66 | B1 | *Fusarium solani* | pith | TDARES | 2020/10/19 |
| 67 | B2 | *Fusarium solani* | pith | TDARES | 2020/10/19 |
| 68 | B6 | *Fusarium solani* | pith | TDARES | 2020/10/19 |
| 69 | C7 | *Fusarium solani* | pith | TDARES | 2020/10/19 |
| 70 | C9 | *Fusarium solani* | pith | TDARES | 2020/10/19 |
| 71 | D7 | *Fusarium solani* | pith | TDARES | 2020/10/19 |
| 72 | D8 | *Fusarium oxysporum* | pith | TDARES | 2020/10/19 |
| **Table S1. continued** | | | | | |
| 73 | D9 | *Fusarium solani* | pith | TDARES | 2020/10/19 |
| 74 | E1 | *Fusarium solani* | pith | TDARES | 2020/10/19 |
| 75 | E4 | *Fusarium solani* | pith | TDARES | 2020/10/19 |
| 76 | E6 | *Fusarium solani* | pith | TDARES | 2020/10/19 |
| 77 | F1 | *Fusarium solani* | pith | TDARES | 2020/10/19 |
| 78 | F2 | *Phomopsis phyllanthicola* | pith | TDARES | 2020/10/19 |
| 79 | F3 | *Fusarium solani* | pith | TDARES | 2020/10/19 |
| 80 | F4 | *Fusarium solani* | pith | TDARES | 2020/10/19 |
| 81 | F5 | *Fusarium solani* | pith | TDARES | 2020/10/19 |
| 82 | F6 | *Fusarium solani* | pith | TDARES | 2020/10/19 |
| 83 | H6 | *Phomopsis* sp. | pith | TDARES | 2020/10/19 |
| 84 | H7 | *Fusarium solani* | pith | TDARES | 2020/10/19 |
| 85 | A9 | *Nigrospora sphaerica* | surface | TDARES | 2020/10/19 |
| 86 | B7 | *Fusarium equiseti* | surface | TDARES | 2020/10/19 |
| 87 | B9 | *Fusarium proliferatum* | surface | TDARES | 2020/10/19 |
| 88 | B10 | *Fusarium equiseti* | surface | TDARES | 2020/10/19 |
| 89 | B11 | *Fusarium solani* | surface | TDARES | 2020/10/19 |
| 90 | B12 | *Fusarium solani* | surface | TDARES | 2020/10/19 |
| 91 | C8 | *Fusarium solani* | surface | TDARES | 2020/10/19 |
| 92 | C10 | *Fusarium solani* | surface | TDARES | 2020/10/19 |
| 93 | C11 | *Fusarium solani* | surface | TDARES | 2020/10/19 |
| 94 | D10 | *Fusarium solani* | surface | TDARES | 2020/10/19 |
| 95 | F8 | *Fusarium solani* | surface | TDARES | 2020/10/19 |
| 96 | F10 | *Fusarium solani* | surface | TDARES | 2020/10/19 |
| 97 | G7 | *Fusarium solani* | surface | TDARES | 2020/10/19 |
| 98 | G8 | *Fusarium equiseti* | surface | TDARES | 2020/10/19 |
| 99 | G9 | *Fusarium equiseti* | surface | TDARES | 2020/10/19 |
| 100 | G10 | *Fusarium solani* | surface | TDARES | 2020/10/19 |
| 101 | G11 | *Fusarium equiseti* | surface | TDARES | 2020/10/19 |
| 102 | G12 | *Fusarium equiseti* | surface | TDARES | 2020/10/19 |
| 103 | H9 | *Fusarium equiseti* | surface | TDARES | 2020/10/19 |
| 104 | H10 | *Fusarium oxysporum* | surface | TDARES | 2020/10/19 |
| 105 | H11 | *Fusarium solani* | surface | TDARES | 2020/10/19 |
| 106 | H12 | *Fusarium solani* | surface | TDARES | 2020/10/19 |
| 107 | C2 | *Fusarium solani* | vascular | TDARES | 2020/10/19 |
| 108 | C3 | *Fusarium solani* | vascular | TDARES | 2020/10/19 |
| 109 | C4 | *Fusarium solani* | vascular | TDARES | 2020/10/19 |
| **Table S1. continued** | | | | | |
| 110 | C6 | *Fusarium solani* | vascular | TDARES | 2020/10/19 |
| 111 | D1 | *Phytopythium vexans* | vascular | TDARES | 2020/10/19 |
| 112 | D3 | *Clonostachys rosea* | vascular | TDARES | 2020/10/19 |
| 113 | E7 | *Glomerella cingulate* | vascular | TDARES | 2020/10/19 |
| 114 | G2 | *Fusarium solani* | vascular | TDARES | 2020/10/19 |
| 115 | G3 | *Fusarium solani* | vascular | TDARES | 2020/10/19 |
| 116 | G4 | *Fusarium solani* | vascular | TDARES | 2020/10/19 |
| 117 | G5 | *Fusarium equiseti* | vascular | TDARES | 2020/10/19 |
| 118 | H1 | *Nigrospora* sp. | vascular | TDARES | 2020/10/19 |
| 119 | H2 | *Diaporthe phaseolorum* | vascular | TDARES | 2020/10/19 |
| ^a^: Species presented in this table were identified with ITS and 16Sr RNA sequences  ^b^: --- denotes data were not recorded  ^c^: National Taitung University, Taitung, Taiwan  ^d^: Taitung District Agricultural Research and Extension Station, Council of Agriculture, Executive Yuan, Taiwan | | | | | |

**Table S2. Sequences and taxonomy of fungal OTUs manually annotated**

| >JX371352_MT251175.1_*Fusarium falciforme*^*^  AAGTCGTAACAAGGTCTCCGTTGGTGAACCAGCGGAGGGATCATTACCGAGTTATACAACTCATCAACCCTGTGAACATACCTATAACGTTGCCTCGGCGGGAACAGACGGCCCCGTAACACGGGCCGCCCCCGCCAGAGGACCCCCTAACTCTGTTTCTATAATGTTTCTTCTGAGTAAACAAGCAAATAAATTAAAACTTTCAACAACGGATCTCTTGGCTCTG  >LC125627_MT925067.1_Uncultured Ascobolaceae  AAGTCGTACAAGGTTTCCGTAGGTGAACCTGCGGAAGGATCATTAAAAAAGTACGCCCCAGCTAAGGTGTAAAACGCTGACTGCTGGTGGCTGTATTCAACCACTTGTTTACTTGTACCTGTTGCTTCCGTGGAGTTACGGGTGCTCACCCTGTTCGCAGGTGTGGGTTACCTTCCACGGGTGTAGTTTGAACGCTTGTTTAGTTGAATGTAGTGTCTGAATAACTATTGTAATAAAGTTAAAACTTTCAACAACGGATCTCTAGGTTCTC  >LT609591_MT924380.1_*Bulleribasidium oberjochense*  AAGTCGTAACAAGGTTTCCGTAGGTGAACCTGCGGAAGGATCATTATTGAATAGACTTTGTTCAAAAGTTTTTTCATATTCCCTGTTTATATTAAATACGTTCCTTCCACTGGACAGGTCTTTCAGGACCCTCTAGCTTAAGCAAATGCTTTTGCTGGGGAGTTTGCCAGTGGCGTTTCTACAAAACAAAAACTTTTGTTATTATGAAATGTCTGAATATATTTTTAAATTAAATAAAACTTTCAACAACGGATCTAGGCTCTT |
| --- |

**^*^**According to the results of BLASTn (NCBI), the names of each fasta sequence in the table were given with a format: fungal OTU ID_accession number_species name

**Table S3. Selected differential abundance of bacterial and fungal genus taxa in healthy and diseased roselle rhizospheres**

|  | Diseased (D) | | | |  | Healthy (H) | | | |
| --- | --- | --- | --- | --- | --- | --- | --- | --- | --- |
|  |  | baseMean | log_2_Fold  Change | *p* value |  |  | baseMean | log_2_Fold  Change | *p* value |
| Bacterial community | g__Microbacterium | 189.1 | -1.6 | 8.26E-07 |  | g__Subgroup_6 | 910.3 | 0.3 | 0.0025 |
|  | g__Luteolibacter | 178.1 | -1.1 | 1.45E-04 |  | g__Saccharimonadales | 357.6 | 0.5 | 0.0024 |
|  | g__Pseudoxanthomonas | 153.4 | -2.4 | 1.17E-05 |  | g__SBR1031 | 139.1 | 0.6 | 0.0009 |
|  | g__Flavobacterium | 148.9 | -1.3 | 5.13E-05 |  | g__0319.6G20 | 129.1 | 0.5 | 7.40E-03 |
|  | g__Achromobacter | 52.3 | -2.2 | 1.87E-05 |  | g__Bacillus | 115.9 | 0.8 | 0.0002 |
|  | g__Peredibacter | 51.7 | -0.7 | 0.0064 |  | g__Subgroup_10 | 94.4 | 0.5 | 0.0047 |
|  | g__Hydrogenophaga | 50.0 | -2.7 | 3.70E-10 |  | g__Gaiella | 91.3 | 0.4 | 0.0031 |
|  | g__Kluyvera | 48.6 | -3.8 | 3.96E-10 |  | g__1959.1 | 85.6 | 2.5 | 1.34E-06 |
|  | g__Cellulosimicrobium | 46.9 | -3.3 | 2.23E-11 |  | g__S0134_terrestrial_group | 63.5 | 0.8 | 0.0038 |
|  | g__Altererythrobacter | 46.5 | -1.6 | 2.96E-05 |  | g__Fimbriimonadaceae | 59.7 | 0.5 | 0.0083 |
| Fungal community | g__Fusarium | 3617.3 | -1.1 | 0.0008 |  | g__Cladorrhinum | 268.0 | 2.0 | 0.0004 |
|  | g__uncultured_Ascobolaceae | 841.9 | -3.2 | 7.77E-07 |  | g__Staphylotrichum | 253.5 | 1.6 | 0.0019 |
|  | g__Myrothecium | 323.0 | -1.4 | 0.0008 |  | g__unidentified_o__Branch06 | 215.2 | 1.5 | 0.00361 |
|  | g__unidentified_o__Hypocreales | 297.7 | -1.8 | 1.13E-06 |  | g__Pseudombrophila | 143.3 | 2.0 | 0.0083 |
|  | g__unidentified_f__Psathyrellaceae | 184.8 | -2.0 | 0.002 |  | g__unidentified_f__Bolbitiaceae | 118.0 | 2.8 | 0.0001 |
|  | g__unidentified_c__Sordariomycetes | 139.4 | -2.2 | 1.40E-05 |  | g__unidentified_f__Chaetomiaceae | 97.3 | 1.5 | 0.0031 |
|  | g__Thanatephorus | 58.6 | -2.7 | 0.0002 |  | g__Neurospora | 84.1 | 2.9 | 1.33E-05 |
|  | g__Conocybe | 31.9 | -1.7 | 0.0054 |  | g__Thielavia | 79.0 | 2.4 | 8.10E-06 |
|  | g__Phyllosticta | 22.4 | -1.7 | 0.0007 |  | g__Acrocalymma | 50.1 | 1.6 | 0.0067 |
|  | g__unidentified_f__Magnaporthaceae | 22.1 | -2.0 | 9.61E-05 |  | g__unidentified_f__Ustilaginaceae | 48.1 | 1.5 | 0.0013 |
